# AIDET communication combined with alternating prone ventilation for HIF-1α, sTREM-1 and hs-CRP in awake elderly patients with severe pneumonia

**DOI:** 10.3389/fmed.2025.1609726

**Published:** 2026-02-04

**Authors:** YanHong Wang, Rong Zhang, XiaoYan Li, Xuejiao Deng

**Affiliations:** Department of Respiratory and Critical Care Medicine, Shanxi Bethune Hospital (Shanxi Academy of Medical Sciences), Taiyuan, Shanxi, China

**Keywords:** AIDET communication, alternating prone ventilation, inflammatory markers, patient outcomes, severe pneumonia

## Abstract

**Background:**

Severe pneumonia in elderly patients carries high mortality, with inflammatory markers such as HIF-1α, sTREM-1, and hs-CRP associated with disease severity. Combining alternating prone ventilation with AIDET communication may improve outcomes.

**Objective:**

To evaluate the effects of AIDET communication combined with alternating prone ventilation on comfort, oxygenation, inflammatory markers, and prognosis in awake elderly patients with severe pneumonia.

**Methods:**

A total of 86 elderly patients were enrolled and assigned to either an AIDET group or a Conventional group (*n* = 43 each). Comfort scores, PaO_2_/FiO_2_ ratios, and serum levels of HIF-1α, sTREM-1, and hs-CRP were assessed at baseline and 72 h. Weaning success and time to withdrawal were also recorded.

**Results:**

At 72 h, the AIDET group demonstrated significantly higher comfort scores (T12: 8.3 ± 0.9 vs. 6.5 ± 1.1; *p* < 0.001) and improved oxygenation (PaO_2_/FiO_2_: 289.4 ± 23.5 vs. 231.2 ± 20.6; *p* < 0.001). Reductions in inflammatory markers were greater in the AIDET group: HIF-1α (↓24.3%; *p* < 0.001), sTREM-1 (↓22.7%; *p* = 0.003), and hs-CRP (↓28.1%; p < 0.001). Weaning success was higher in the AIDET group (95.3% vs. 76.7%; *p* = 0.013) with a shorter median time to weaning (47 h vs. 62 h; *p* < 0.001).

**Conclusion:**

In elderly patients with severe pneumonia, AIDET communication combined with prone ventilation significantly improves comfort, oxygenation, and biomarker profiles, and enhances weaning success. These findings support the integration of structured communication into respiratory care protocols.

## Introduction

With the rapid aging of populations worldwide, the incidence of severe community-acquired pneumonia (CAP) among older adults continues to rise. This condition carries high morbidity and mortality, posing significant challenges to healthcare systems globally (1). Its high morbidity and mortality rates have posed huge challenges to China public health system. The causative bacteria of elderly CAP are mostly mixed infections, and the clinical symptoms are often nonspecific or atypical (2). Patients are often affected by multiple comorbidities, impaired immune function, and reduced ability to perform daily activities (3), clinical treatment and management are complex. Airway management is a key approach in the treatment of severe CAP in the elderly (4). Prone position ventilation can reduce the intrathoracic pressure gradient, promote secretion drainage and intrapulmonary fluid movement, and significantly improve oxygenation levels. It has increasingly been adopted as an airway management strategy for patients with respiratory distress syndrome (5) and viral pneumonia (6). Xu et al. (7) found that prone position ventilation in the ICU can reduce the detection rate of multi-drug-resistant bacteria in pneumonia, and reduce mechanical ventilation time and improve patient retrieval outcomes and length of hospitalization. This indicates that prone position ventilation is an effective airway management strategy and is beneficial to improving the oxygenation status of elderly patients with severe CAP.

Improvement of oxygenation status is beneficial to the repair of pulmonary air-blood barrier (Alveolar-capillary barrier) function. Hypoxia-inducible factor 1 (HIF-1α) is a heterodimeric transcription factor consisting of two subunits, HIF-1α and HIF-1β (8). Hypoxia associated with pneumonia patients increases HIF-1α stability, induces activation of hypoxic target genes, and promotes inflammatory factor production (9, 10). Additionally, intracellular HIF-1α levels may influence sTREM-1 expression (11). sTREM-1, as an inflammatory marker, has been shown to be closely associated with the severity and prognosis of severe pneumonia in the elderly, especially in patients with bacterial pneumonia and critical pneumonia (12–15). hs-CRP, as a blood marker of inflammation, has been commonly used to assess the organism’s inflammation level and degree of infection (16). Variations in hs-CRP levels may reflect treatment response, making it a useful biomarker for monitoring therapeutic efficacy during prone ventilation (17). By monitoring hs-CRP levels, physicians can assess the patient’s disease progression and guide airway management and adjustment of treatment regimens.

Clinically, patient compliance during prone position is directly related to the outcome of oxygenation improvement, however, elderly patients with severe CAP have incidents of non-cooperation in completing prone ventilation even in the awake state. The AIDET communication model is an effective way to enhance the degree of patients’ cooperation in treatment, which can improve patients’ oxygenation and clinical prognosis (18). Some scholars have applied it to patients with pneumonia on alternating prone ventilation and established an effective communication process, but whether this method is applicable to elderly awake patients still needs to be further studied (19). Regular monitoring of hs-CRP levels during prone ventilation may help to adjust the treatment plan in time. However, at present, whether elderly patients with severe pneumonia can produce further improvements in sTREM-1 and hs-CRP based on improved oxygenation indices by applying the optimized form of prone position ventilation needs further study.

In addition to HIF-1α, sTREM-1, and hs-CRP, sTREM-1 was also evaluated in this study due to its critical role in leukocyte-endothelial adhesion and transmigration during pulmonary inflammation. sTREM-1 is the circulating form of sTREM-1, an adhesion molecule expressed on vascular endothelial cells during inflammation. Its elevation has been associated with endothelial activation and lung injury severity in patients with pneumonia and ARDS (20). Monitoring sTREM-1 levels may offer additional insight into the vascular component of the inflammatory response, particularly in elderly patients with impaired microcirculation and comorbidities. Thus, it was included as a secondary biomarker to complement the understanding of systemic inflammation in this patient population.

Hence, this study hypothesizes that elderly patients with severe CAP can use the AIDET communication mode combined with alternating prone position ventilation to improve their oxygenation status, which in turn suppresses the body’s inflammatory response, reduces sTREM-1, hs-CRP levels, and subsequently improves clinical prognosis.

## Materials and methods

### General data

This was a prospective, comparative observational study conducted at Shanxi Bethune Hospital between March 2020 and April 2023. A total of 86 elderly patients meeting the inclusion criteria were enrolled and divided into two groups: the AIDET group (*n* = 43) and the Conventional group (*n* = 43). Group assignment was performed using a simple alternation approach based on admission sequence or availability of personnel trained in the AIDET communication protocol. No formal randomization or allocation concealment was applied. The study did not involve blinding due to the nature of the intervention.

### Sample size consideration

Due to the pragmatic and exploratory nature of this prospective observational study, a formal *a priori* sample size calculation or statistical power analysis was not performed. The final sample size of 86 patients (43 per group) was determined based on patient availability over the 3-year enrollment period and logistical feasibility within the study setting, using convenient sampling technique. Although this limited sample size may restrict the generalizability of findings and the detection of small effect sizes, statistically significant differences were observed across key outcomes, including oxygenation index, inflammatory biomarkers (HIF-1α, sTREM-1, hs-CRP), and weaning success.

#### Inclusion criteria

(1) PaO_2_/FiO_2_ is continuously lower than 150 mmHg (1 mmHg = 0.133 kPa), blood oxygen saturation is <90%; (2) blood oxygen saturation is <90%, and nasal high-flow humidified oxygen inhalation or non-invasive ventilator is used the oxygen concentration is 30–60%. When the oxygen flow reaches 2–10 L/min through a nasal cannula or mask, the blood oxygen saturation is greater than 94%; (3) Clear consciousness, tolerance to the prone position, and the ability to change positions independently.

#### Exclusion criteria

(1) Patients with intracranial hypertension, severe arrhythmia, and hemodynamic instability; (2) Patients with limb dysfunction, associated fractures or disabilities; (3) Patients with abdominal trauma who cannot tolerate prone position ventilation; (4) Patients refuse or interrupt participation this study. (5) Those with incomplete clinical data. Conventional group: 26 males and 17 females; aged 65 to 85 years old, average age (72.66 ± 4.75) years; BMI 18.7 ~ 24.2 kg/m^2^, average (22.14 ± 1.35) kg/m^2^; acute physiological and chronic health conditions Score II (acute physiology and chronic health evaluation II, APACHE II) 14 to 22 points, average (18.43 ± 1.69) points; clinical pulmonary infection score (CPIS) 6 to 12 points, average (7.96 ± 0.41) points. AIDET group: 28 males and 15 females; age 65–84 years old, average age (73.15 ± 5.03) years; BMI 18.7 ~ 24.2 kg/m2, average (22.75 ± 1.63) kg/m^2^; APACHEII score 15 ~ 22 points, The average score was (18.50 ± 1.76); the CPIS score was 6 to 12 points, with an average score of (7.96 ± 0.57). There was no statistically significant difference in gender, age, APACHEII and other data between the two groups of patients (*p* > 0.05), and the comparability was strong.

### Procedure

Both groups received standard care, including ECG monitoring, phlegm reduction, anti-infection measures, and symptomatic treatment.

### Conventional group

The Conventional group received standard invasive mechanical ventilation in the prone position. The ventilation settings were as follows: tidal volume of 8–10 mL/kg, pressure support of 8–40 cmH2O, and a respiratory rate of 8–12 breaths per minute. Patients were assisted into the prone position with their head down and feet elevated. Feeding and eating were stopped 30 min before ventilation to avoid reflux. During treatment, the patient’s arm, ankle, knee, head, face, iliac, and shoulder positions were adjusted to ensure comfort. Soft pads were placed as needed, and position changes were conducted every 1 to 2 h. Upper limbs were restrained when necessary to prevent accidental extubation.

### AIDET group

**Figure 1:**
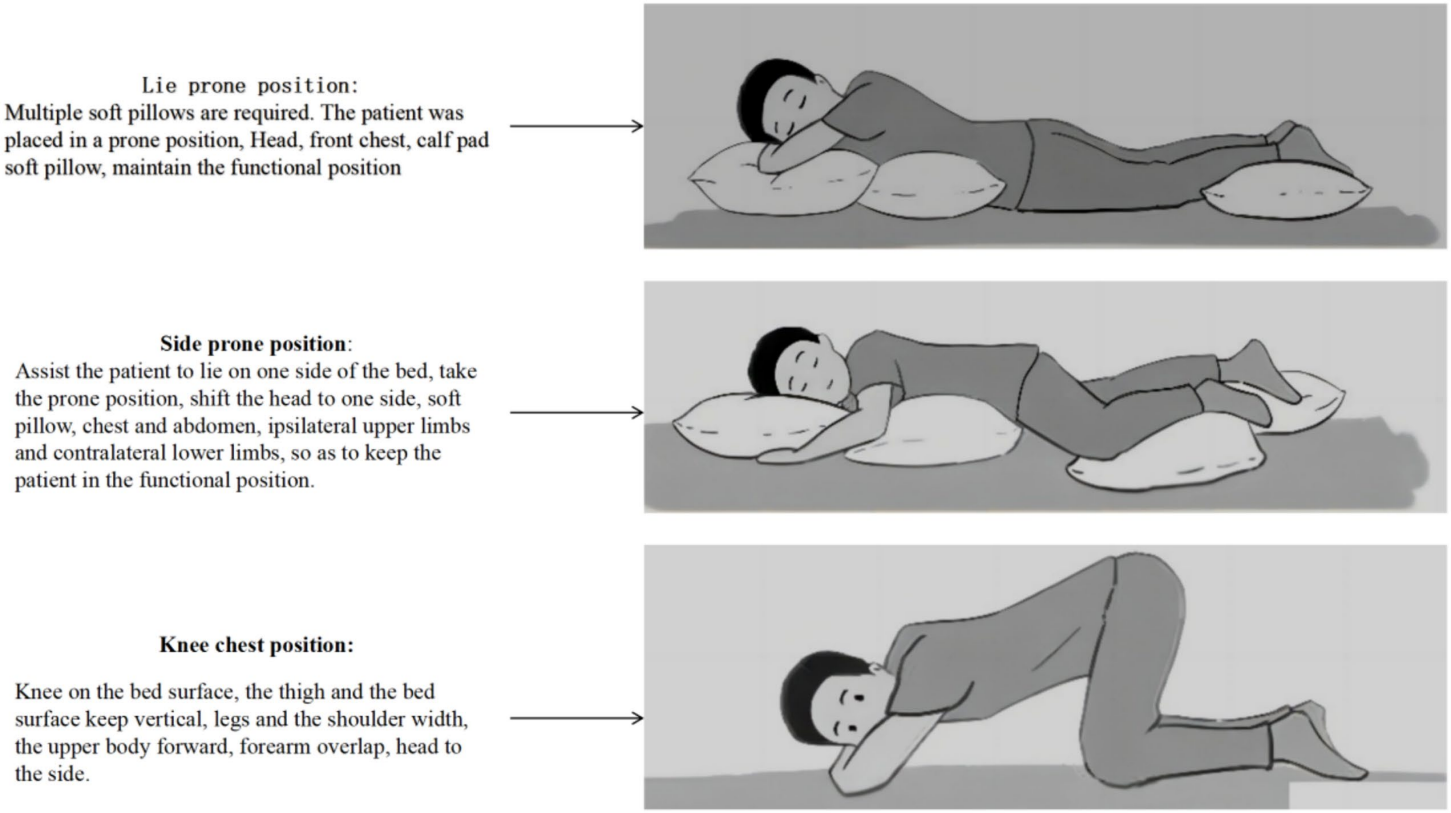
Schematic diagram of the prone ventilation position in the AIDET group.

AIDET communication and alternating prone ventilation treatment were used. (1) Establish an intervention team: Establish a multidisciplinary collaborative team for prone position ventilation treatment. The six core members of the multidisciplinary collaborative team include a critical care physician, head nurse, quality control team leader, accelerated recovery specialist nurse, and respiratory therapy specialist nurse, rehabilitation therapist, and other team members are the doctor in charge and the responsible nurse. Core members are responsible for program formulation, implementation and quality control. The doctor in charge and the rehabilitation therapist are mainly responsible for evaluating and determining the specific plan for prone position ventilation for patients; the head nurse and specialist nurses are responsible for quality control, feedback, and improvement of the implementation of the plan; and the responsible nurse is responsible for the implementation of the plan. (2) Formulate an alternating prone position ventilation plan: refer to the guidelines or consensus literature key points of RAOOF et al. (20), and based on evidence and combined with the clinical characteristics of elderly patients with severe pneumonia, formulate an AIDET communication model suitable for alternating prone position ventilation. The specific content is: A (Acknowledge): Greet the patient cordially, check the patient’s information, smile, and have a good attitude; considering that elderly patients may have hearing loss or cognitive function decline, they should speak slower and more clearly. Greet in the appropriate language and make sure there is adequate eye contact. And evaluate whether the patient has worries, uneasiness, anxiety and other emotions through language, behavior, expression, etc., and provide timely comfort and encouragement. I (Introduce): Elderly patients may not be familiar with medical terminology, so they need to use simpler, more life-like language when introducing, and repeat key information multiple times to enhance understanding. Explain to the patient in simple and easy-to-understand language the purpose and expected effects of alternating prone ventilation, as well as the matters that the patient needs to cooperate with. He also introduced in detail the clinical experience and professional experience of his team’s doctors and respiratory therapists to build patients’ confidence in cooperating with the medical team for treatment. D (Duration): Elderly patients have reduced physiological reserves and may have poor tolerance for prone position ventilation. A more detailed assessment of the patient’s overall condition, including cardiopulmonary function, skin condition, psychological status, nutritional status, and catheters and lines, is needed to ensure the safety of position changes during prone position ventilation. Assess the patient’s vital signs, oxygen therapy method, and whether the respiratory tract is unobstructed, and promptly remove secretions from the mouth, nose, and airways. Assess the skin at the pressure area, use foam accessories locally to reduce pressure, and assess the direction of turning in the prone position. E (Explanation): Explain the steps and precautions for prone position ventilation to the patient in detail, including how to cooperate with medical staff to flip the position. Make preparations before turning over, sort out various wires, drainage tubes, and infusion channels, clamp or remove non-emergency lines, and secure each catheter properly to prevent it from falling off. Align the direction of each catheter with the longitudinal axis of the body, and leave enough length for easy flipping. After confirming that the patient is able to perform the glute bridge exercise, the buttocks are lifted off the bed. Instruct older patients to understand and remember information related to the prone ventilation position through repetition and reinforcement. Use illustrations, demonstrations and other auxiliary means to help them understand the process and importance of prone position ventilation, and provide patients with pictures showing the prone position ventilation posture, see Figure 1 for details. Patients can freely choose 3 positions (prone position, chest and knee position, side prone position) for alternating prone position ventilation. Remove the electrode pads before prone positioning, and attach the electrode pads to the back while lying prone. Keep your head high and your feet low when lying prone to reduce head and face edema. Carry out prone position ventilation according to the specific measures in the plan, exclude eating and going out for examination and treatment, and extend the prone position ventilation time as much as possible. During the position change process, a rehabilitation therapist or senior nurse serves as the team leader, standing on the side of the patient’s head, responsible for issuing instructions and observing the patient’s reaction during the turning process, listening to the chief complaint, and evaluating various indicators. The other two people stood on both sides of the bed. One person is responsible for assessing whether the patient’s wires, pipes, infusion channels, etc. are unobstructed and in functional positions; one person is responsible for assisting the patient in prone positioning and placement of decompression tools. The team leader instructs the patient to turn to one side to complete the posture, and observes the patient at the bedside for 15 min to ensure that the patient’s position is comfortable. During the evaluation period, lung buckling or lung concussion decontamination treatment is performed. Patients who are obese or have weak mobility can be turned over by 4 to 5 people. Actively ask the patient if he or she has any doubts, encourage them to express them and patiently answer them. T (Thank you): After each treatment, thank the patient for his cooperation and efforts. Elderly patients may be worried about their health, and thanking them for their courage and efforts can boost their self-esteem and confidence. Prone ventilation was stopped when the patient failed to improve the oxygenation index or developed serious complications; the oxygenation index decreased compared with that in the flat position; the respiratory rate was greater than 40 breaths/min; the haemodynamics was unstable, very uncomfortable, and could not tolerate the prone position. The withdrawal operation was started when the patient met the conditions for withdrawal.

### Staff training and fidelity monitoring

To ensure standardized implementation of the AIDET communication model, all healthcare personnel involved in the intervention—critical care physicians, nurses, and respiratory therapists—underwent structured training before the study commenced. The training program included a 4-h workshop led by certified clinical educators and a senior nurse educator, covering the AIDET components (Acknowledge, Introduce, Duration, Explanation, Thank you), communication role-play, and case-based simulations with elderly patients. Staff were required to demonstrate competency through supervised practice sessions and a checklist-based evaluation. Throughout the study, intervention fidelity was monitored via direct observations and weekly audits by the head nurse and quality control officer. Adherence to the AIDET protocol was assessed using a standardized compliance checklist (covering verbal communication structure, patient engagement, and explanation quality). Inter-rater reliability for fidelity scoring was established between two independent observers (Cohen’s kappa = 0.81). Feedback was provided in real time to staff to ensure consistent protocol delivery across all shifts. Deviations were logged and addressed through targeted retraining sessions when needed. These measures aimed to minimize variability in the execution of the AIDET communication model and strengthen internal validity.

### Observation index

#### The comfort level

Adopt the analog digital scoring method (0 to 10 points), and the patients will score according to their own comfort level. The higher the score, the higher the comfort level. Collect patients; daily comfort scores for comparison. For each patient, the time points before ventilation (T0), 1 h of ventilation (T1), 6 h of ventilation (T6), and 12 h of ventilation (T12) were taken as the comfort investigation time points.

#### Changes in oxygenation index

The value of arterial blood oxygenation index PaO_2_/FiO_2_ was measured using blood gas analysis before prone position ventilation (T0), 24 h (T24), and 72 h (T72) of prone position ventilation.

#### Serological indicators

Serum levels of HIF-1α, sTREM-1, and hs-CRP were assessed at baseline (T0) and after 72 h of intervention (T72). sTREM-1 was included to evaluate endothelial activation and leukocyte trafficking, which contribute to lung tissue injury and systemic inflammation. HIF-1α and sTREM-1 were measured using enzyme-linked immunosorbent assay (ELISA), while hs-CRP was measured via immunoturbidimetry.

### Prognosis

The weaning time and success of weaning were collected from the two groups of patients. The observation time was set to 72 h, and the weaning time was from the time when the patients ventilation started to the time of successful weaning. Successful weaning follows the patient’s recovery of spontaneous breathing, good oxygenation, and no significant increase in PaCO_2_, decrease in PaO_2_, increased respiratory rate, increased heart rate, extreme fatigue, or excessive sternocleidomastoid muscle movement within 48 h after weaning from the ventilator, heart failure, arrhythmia, shock or worsening of consciousness and other symptoms.

### Statistical methods

GraphPad Prism 10 software was used for data processing, $\bar{x}\pm s$ descriptions were applied to the measurement data, independent samples *t*-test was used for comparison between groups, paired samples t-test was used for comparison within groups at different times, non-normally distributed data with variance chi-square was used for non-parametric rank sum test, qualitative data was expressed as n (%), and the chi-square test was applied with the correction level of *α* = 0.05. Survival curve analysis was used to determine the success of the machine withdrawal in the two groups. Time difference.

## Results

### Baseline characteristics

Table 1 presents the baseline demographic and clinical characteristics of patients in the AIDET and Conventional groups. No statistically significant differences were observed between the two groups in terms of age, sex distribution, BMI, APACHE II score, or CPIS score (*p* > 0.05), indicating baseline comparability.

**Table 1:** Baseline characteristics of patients in AIDET vs. conventional group.

Variable	AIDET group (*n* = 43)	Conventional group (*n* = 43)	*p*-value
Age (years), mean ± SD	73.15 ± 5.03	72.66 ± 4.75	0.587^1^
Sex, *n* (%)			0.654^2^
Male	28 (65.1%)	26 (60.5%)	
Female	15 (34.9%)	17 (39.5%)	
BMI (kg/m^2^), mean ± SD	22.75 ± 1.63	22.14 ± 1.35	0.108^1^
APACHE II score, mean ± SD	18.50 ± 1.76	18.43 ± 1.69	0.833^1^
CPIS score, mean ± SD	7.96 ± 0.57	7.96 ± 0.41	0.996^1^

1 Independent samples *t*-test.

2 Chi-square test.

### Changes in the comfort level of patients in two groups

**Figure 2:**
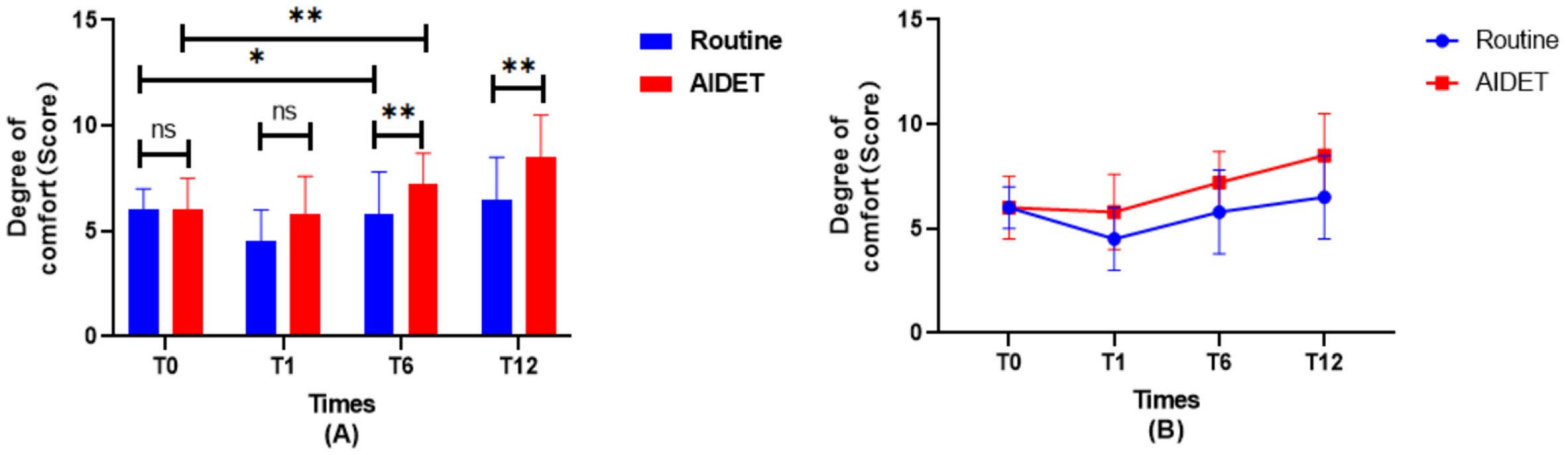
Comparison of changes in patient comfort between the two groups. **(A)** is a bar graph comparing the comfort level of patients in the two groups; **(B)** is a line graph of the comfort level of patients in the two groups. T0 is before ventilation, T1 is ventilation for 1 h, T6 is ventilation for 6 h, and T12 is ventilation for 12 h.

As seen in Figure 2A, there was no statistical significance in the comparison of the comfort level of patients in the two groups before ventilation and 1 h of ventilation, and the comfort level of patients in 6 h of ventilation (T6) and 12 h of ventilation (T12) increased, and the AIDET group was higher than the Conventional group, and the difference was statistically significant (*p* < 0.05). As seen in Figure 2B, the AIDET group was located above the Conventional group at all 3 time points after ventilation.

### Comparison of PaO_2_/FiO_2_

**Figure 3:**
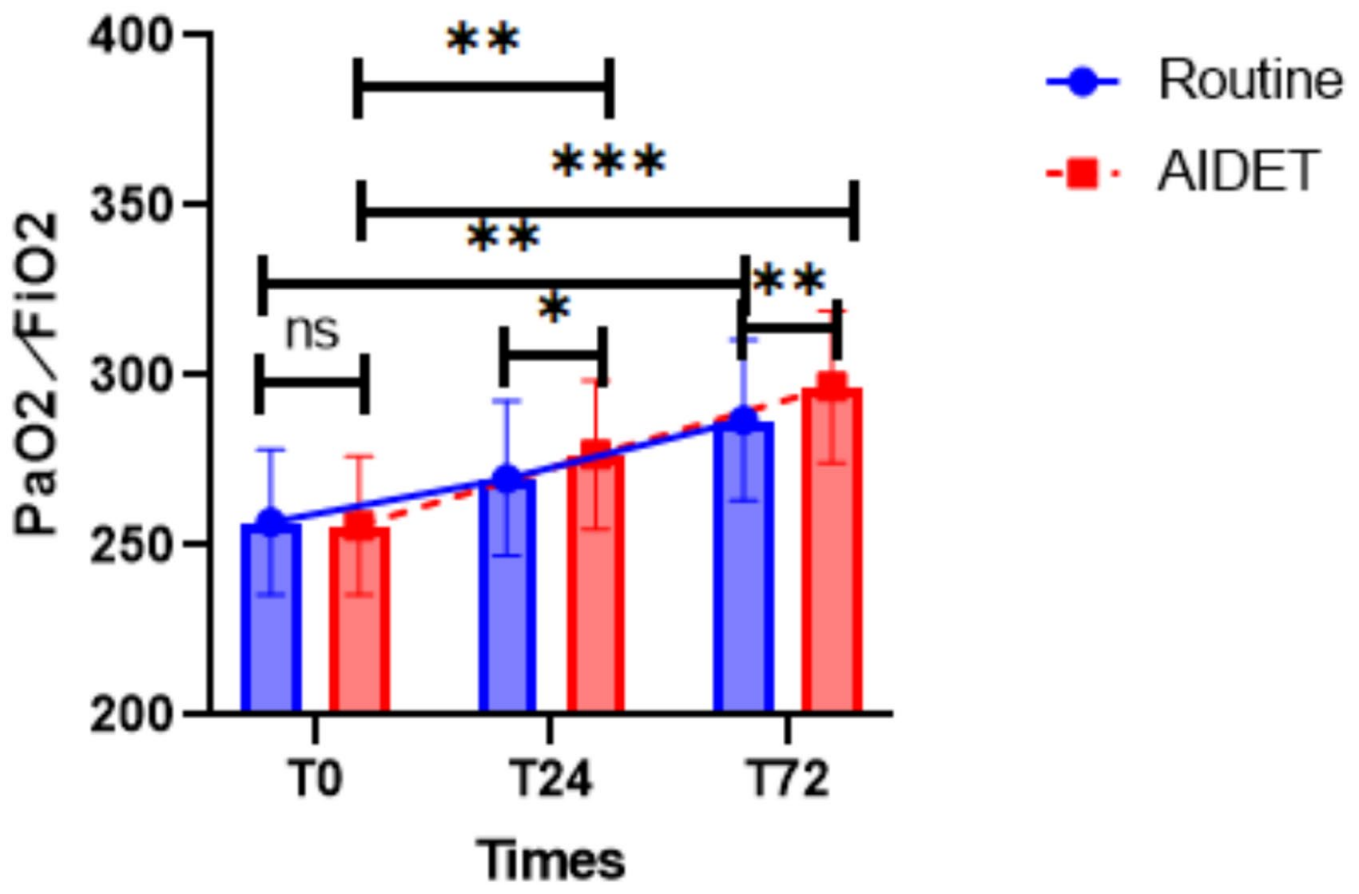
Comparison of changes in oxygenation index between the two groups of patients. Conventional is the Conventional group, AIDET is the AIDET group, T0 is pre-ventilation, T24 is ventilation for 24 h, and T72 is ventilation for 72 h.

As seen in Figure 3, there was no statistical significance in the comparison of PaO_2_/FiO_2_ between the two groups before ventilation (T0), and the two groups of prone position ventilation for 24 h (T24) and ventilation for 72 h (T72) improved compared with the preventive period, but the AIDET group’s PaO_2_/FiO_2_ was significantly higher than the Conventional group, and the difference was statistically significant (*p* < 0.05).

### HIF-1α, sTREM-1, hs-CRP levels of patients in two groups

**Figure 4:**
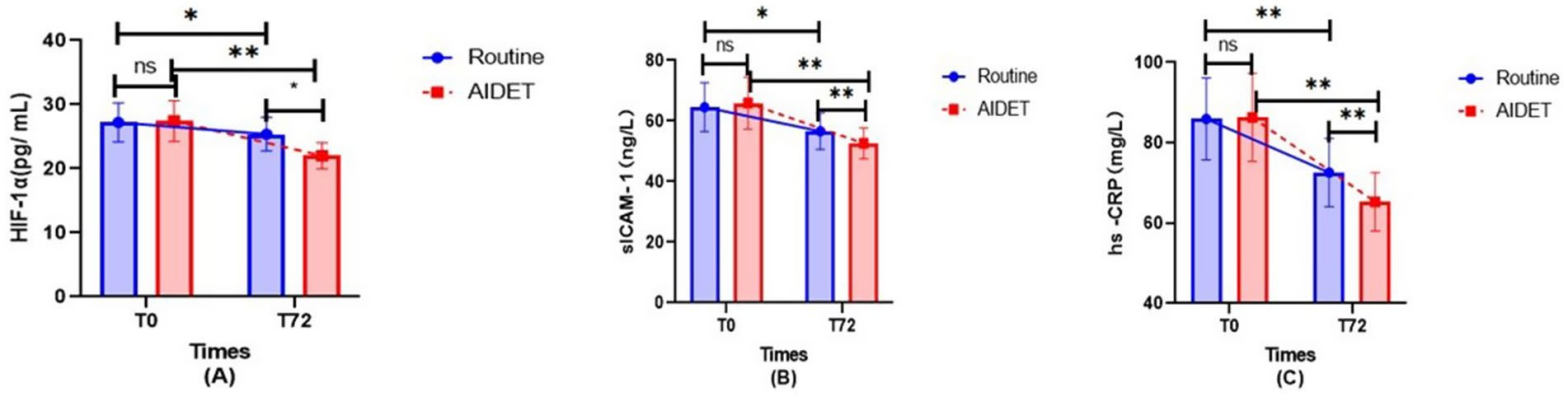
Comparison of the changes of HIF-1α, sTREM- 1, and hs -CRP between the two groups of patients. **(A)** Hypoxia-inducible factor-1α (HIF-1α, pg/mL). **(B)** Soluble intercellular adhesion molecule-1 (sICAM-1, ng/L). **(C)** High-sensitivity C-reactive protein (hs-CRP, mg/L).

There was no statistically significant difference in HIF-1α, sTREM-1, and hs-CRP levels between the two groups before ventilation (*p* > 0.05); the levels of HIF-1α, sTREM-1, and hs-CRP in the AIDET group at 72 h of ventilation (T72) were decreased compared with those in the Conventional group and were lower than those before ventilation in this group, and the difference was statistically significant (*p* < 0.05). See Figure 4 for details.

### Comparison of the prognosis of patients in the two groups

**Figure 5:**
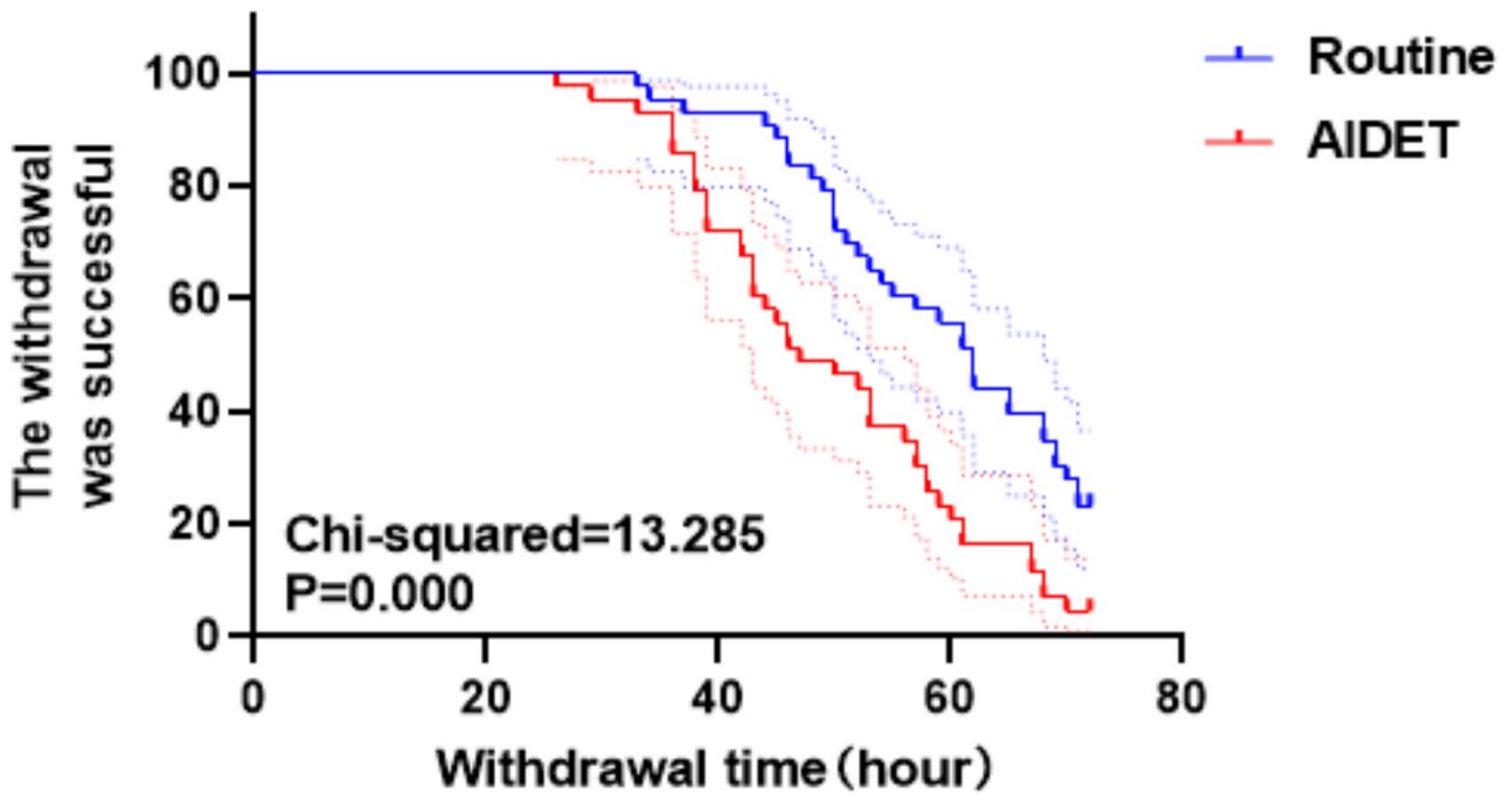
Successful curves of machine withdrawal for both patients in both groups. The blue line is the Conventional group, the red line is the AIDET group, and the line decreases as the withdrawal time increases, with a higher decrease representing a higher success rate of patient withdrawal.

The withdrawal success rate in the AIDET group was 95.35% (41/43), significantly higher than the 76.74% (33/43) observed in the Conventional group, and the difference was statistically significant (Chi-squared = 13.285, *p* = 0.000) (Figure 5).

## Discussion

This study focuses on the application of AIDET communication mode and alternating prone ventilation in awake elderly patients with severe pneumonia. The study was conducted on 86 elderly conscious patients with severe pneumonia were randomly divided into a Conventional group and an AIDET group. The Conventional group used conventional prone ventilation, while the AIDET group applied AIDET communication mode combined with alternating prone ventilation on this basis. The main observation indicators were patient comfort, PaO_2_/FiO_2_, HIF-1α, sTREM-1, hs-CRP, weaning success rate and weaning time. The results suggest that the AIDET communication model combined with alternating prone position ventilation may be an effective treatment strategy, which can improve the comfort and oxygenation index of awake elderly patients with severe pneumonia, reduce the inflammatory response, and help improve the success rate of weaning and shorten the time of weaning. Weaning time. These findings have important reference value for clinical medical staff when formulating treatment plans.

Severe pneumonia in the elderly is a common clinical critical illness, which is characterized by rapid disease progression, often accompanied by multiple organ dysfunction and high mortality (21). HIF-1α is a specific transcriptional regulator that cells produce in response to hypoxic environment when stimulated by ischemia and hypoxia (22). Severe pneumonia is typically accompanied by lung injury, with overlapping processes of inflammation and hypoxia. Inflammation and Hypoxia interacts with each other, affecting the gas exchange function of the lungs, leading to a rapid decrease in oxygen partial pressure, causing the body to undergo a hypoxic reaction, increasing the expression of HIF-1α and participating in the occurrence and development of lung injury (22). sTREM-l serves as a The product of ICAM-1 hydrolysis on the surface of seed cells is a new type of inflammatory transmitter, which has the function of regulating the adhesion between leukocytes and endothelial cells, promoting the migration of leukocytes, and amplifying the cascade of inflammatory responses (20). hs-CRP is a non-specific inflammatory marker that can exert a pro-inflammatory effect by inducing the release of inflammatory cytokines such as TNF-*α* and IL-6 (23); it can also activate platelets and enhance platelet metabolism. The adhesion and aggregation function damages vascular endothelial cells and causes microcirculation disorders in important organs and tissues. At the same time, cytokines and chemical mediators released during inflammation, such as prostaglandins and leukotrienes, can increase vascular permeability, leading to local swelling, heat, and pain. These symptoms reduce patient comfort. AIDET, as a person-centered communication strategy, has been proven to improve patient comfort and treatment effectiveness (24). As an effective means of respiratory support, alternating prone position ventilation plays an important role in improving oxygenation and reducing mortality.

The results of this study compare favorably with other studies, but there are some unique findings. For example, several previous studies have demonstrated that AIDET improves patient satisfaction and comfort (25) and is a continuous quality improvement tool in emergency medicine (18, 26, 27). Yang et al. (28) scholars used the AIDET standard communication model for pregnant women and nurses to promote health education together and were able to reduce the level of postoperative anxiety in pregnant women. Some scholars even used it for patients undergoing percutaneous coronary intervention and found that this modality was able to improve patients’ prognosis (29). Prone position ventilation improves the oxygenation status of patients with ARDS, which is consistent with our observation of an improved PaO_2_/FiO_2_ ratio in the AIDET group. However, our study further found that the improvement in oxygenation due to prone position ventilation was more significant with the assistance of the AIDET communication mode, a finding that has not been widely reported in the existing literature. In addition, our study showed that AIDET communication with prone position ventilation was able to reduce the inflammatory response in elderly patients with severe pneumonia, which was demonstrated by the decrease in HIF-1α, sTREM-1, and hs-CRP levels. Although, the role of inflammatory markers in the assessment of disease severity is widely recognized, the specific effect of AIDET communication mode on inflammatory marker levels needs to be elucidated by more studies.

In terms of withdrawal success rate and time to withdrawal, our study showed that the AIDET group had a higher withdrawal success rate and shorter time to withdrawal, which is considered to be related to the AIDET communication model improving overall patient comfort and engagement. This has been widely recognized in previous studies and therefore our study provides new insights into understanding the role of patient communication in the withdrawal process.

### Limitations

Despite the promising findings, this study has several limitations. First, the relatively small sample size and single-center design limit the generalizability of the results and may reduce statistical power to detect smaller but clinically relevant differences. Second, the absence of formal randomization and allocation concealment introduces the possibility of selection bias, and no stratification was performed based on baseline severity or comorbidities. Third, although baseline characteristics appeared comparable between groups, residual confounding from unmeasured variables (e.g., medication history, nutritional status, or underlying frailty) cannot be ruled out. Fourth, adherence to the prone ventilation protocol and fidelity to the AIDET communication framework may have varied among staff members, which could affect intervention consistency. Fifth, the study focused on short-term outcomes (72 h), and longer-term effects on survival, readmission, or lung function were not assessed. Finally, the study lacked blinding for patients and clinicians, which may have influenced subjective measures such as comfort scores. These limitations highlight the need for larger, multicenter randomized trials to validate the present findings and explore their long-term implications. Although fidelity monitoring and staff training were performed, complete standardization of human communication is inherently difficult. Minor variability in communication tone, experience level, and adherence may have influenced outcomes despite control measures.

Another important limitation of this study is its bundled intervention design. The AIDET communication model and alternating prone ventilation were introduced simultaneously in the intervention group, without separate control arms for each component. As a result, the individual contributions of AIDET communication and prone positioning to the observed improvements in oxygenation, comfort, inflammatory markers, and weaning success could not be disentangled. Future studies should consider a factorial or multi-arm randomized design to isolate and compare the specific effects of communication models and physical interventions such as prone positioning.

Another limitation of this study is the restricted temporal resolution of biomarker measurement. Inflammatory markers (HIF-1α, sTREM-1, and hs-CRP) were measured only at baseline (T0) and at 72 h (T72). This sampling strategy was chosen based on logistical feasibility and the assumption that 72 h would reflect early inflammatory response to the intervention. However, the lack of intermediate time points (e.g., 24 h or 48 h) prevents us from understanding the precise kinetics of biomarker changes—whether they declined steadily, peaked earlier, or were influenced by short-term clinical fluctuations.

The use of subjective measures such as self-reported comfort scores introduces the potential for reporting bias, particularly as no geriatric-specific validation of the analog scale was performed. Additionally, while “weaning success” was operationally defined using physiological criteria, interpretation may vary by clinical assessor, and the absence of assessor blinding could have introduced bias.

## Conclusion

In this study, we have thoroughly explored the effectiveness of the application of the AIDET communication mode combined with alternating prone position ventilation in the treatment of elderly awake patients with severe pneumonia. The results of the study showed that the application of the AIDET communication mode significantly enhanced patient comfort and significantly improved the PaO_2_/FiO_2_ ratio at 24 and 72 h of ventilation, reflecting an improvement in the patients’ oxygenation status. In addition, the levels of inflammatory markers HIF-1α, sTREM-1, and hs-CRP in the AIDET group at 72 h of ventilation decreased significantly compared with those in the Conventional group, which not only revealed the possible modulating effect of the AIDET communication mode on the inflammatory response, but also highlighted its potential value in the assessment of disease severity.

## Data Availability

The original contributions presented in the study are included in the article/supplementary material, further inquiries can be directed to the corresponding author.
